# Serum immuno-oncology markers carry independent prognostic information in patients with newly diagnosed metastatic breast cancer, from a prospective observational study

**DOI:** 10.1186/s13058-023-01631-6

**Published:** 2023-03-21

**Authors:** Frida Björk Gunnarsdottir, Pär-Ola Bendahl, Alexandra Johansson, Rui Benfeitas, Lisa Rydén, Caroline Bergenfelz, Anna-Maria Larsson

**Affiliations:** 1 Division of Experimental Infection Medicine, Department of Translational Medicine, Lund University, SE-214 28 Malmö, Sweden; 2 Division of Oncology, Department of Clinical Sciences Lund, Lund University, SE-223 81 Lund, Sweden; 3 National Bioinformatics Infrastructure Sweden (NBIS), Science for Life Laboratory, Department of Biochemistry and Biophysics, Stockholm University, SE-106 91 Stockholm, Sweden; 4 Department of Surgery and Gastroenterology, Skåne University Hospital, SE-214 28 Malmö, Sweden

**Keywords:** Serum, Immuno-oncology, Marker, Metastatic breast cancer, Survival

## Abstract

**Background:**

Metastatic breast cancer (MBC) is a challenging disease, and despite new therapies, prognosis is still poor for a majority of patients. There is a clinical need for improved prognostication where immuno-oncology markers can provide important information. The aim of this study was to evaluate serum immuno-oncology markers in MBC patients and their respective relevance for prediction of survival.

**Patients and methods:**

We investigated a broad panel of 92 immuno-oncology proteins in serum from 136 MBC patients included in a prospective observational study (NCT01322893) with long-term follow-up. Serum samples were collected before start of systemic therapy and analyzed using multiplex proximity extension assay (Olink Target 96 Immuno-Oncology panel). Multiple machine learning techniques were used to identify serum markers with highest importance for prediction of overall and progression-free survival (OS and PFS), and associations to survival were further evaluated using Cox regression analyses. False discovery rate was then used to adjust for multiple comparisons.

**Results:**

Using random forest and random survival forest analyses, we identified the top nine and ten variables of highest predictive importance for OS and PFS, respectively. Cox regression analyses revealed significant associations (*P* < 0.005) of higher serum levels of IL-8, IL-10 and CAIX with worse OS in multivariable analyses, adjusted for established clinical prognostic factors including circulating tumor cells (CTCs). Similarly, high serum levels of IL-8, IL-10, ADA and CASP8 significantly associated with worse PFS. Interestingly, high serum levels of FasL significantly associated with improved OS and PFS. In addition, CSF-1, IL-6, MUC16, TFNSFR4 and CD244 showed suggestive evidence (*P* < 0.05) for an association to survival in multivariable analyses. After correction for multiple comparisons, IL-8 still showed strong evidence for correlation to survival.

**Conclusion:**

To conclude, we found six serum immuno-oncology markers that were significantly associated with OS and/or PFS in MBC patients, independently of other established prognostic factors including CTCs. Furthermore, an additional five serum immuno-oncology markers provided suggestive evidence for an independent association to survival. These findings highlight the relevance of immuno-oncology serum markers in MBC patients and support their usefulness for improved prognostication.

*Trial registration* Clinical Trials (NCT01322893), registered March 25, 2011.

**Supplementary Information:**

The online version contains supplementary material available at 10.1186/s13058-023-01631-6.

## Background

While early-stage breast cancer (BC) has a good prognosis for most patients, advanced or metastatic breast cancer (MBC) is generally considered to be an incurable disease. MBC treatment is complex and used for alleviation of symptoms and improving quality of life of patients, as well as extending survival. Median overall survival (OS) for MBC patients is around three years, with a 5-year survival rate of approximately 25% [1]. Even though treatment of MBC has improved, there is an urgent need for biomarkers that could be used to improve prognostication and treatment prediction as well as to monitor therapy response to better individualize treatment. Liquid biopsies, including serum markers, have potential to contribute to improved tailoring of systemic therapy and are easily accessible via regular blood samples.

Evaluation of blood-borne markers in cancer patients has been gaining attention, but most studies on multiple protein biomarkers in blood from BC patients have not looked specifically at MBC, but rather compared serum proteins from BC patients at different BC stages and healthy controls, or from patients before and after treatment [2–4]. Many studies have further focused on specific chemokines or tumor-derived markers, rather than a wide panel of cytokines, chemokines, and tumor-related proteins [5, 6]. Most previous studies that have investigated prognostic biomarkers in MBC have also focused on a very limited panel of markers [7–10].

With newly available multi-screening methods, it is now possible to measure hundreds of markers in smaller amounts of blood, serum or plasma than before [11]. One such method is proximity extension assay (PEA), where antibodies that are linked to oligonucleotides are pair-bound and their oligonucleotides hybridized and quantified with real-time quantitative polymerase chain reaction (RT-qPCR) upon binding to target proteins. This allows for simultaneous relative quantification of 92 proteins in small volumes of serum or plasma with high specificity and sensitivity [11]. Studies that have used PEA assay panels in research on breast cancer patients have so far only looked at early BC, treatment response, or have not used blood but rather fine-needle aspirations directly from breast cancer tumors or microdialysis of breast tissue [12–15]. Hence, evaluation of multiple serum proteins in MBC patients and their potential value in prognostication and therapy monitoring is sparse.

BC has historically been considered immunologically “cold”. Yet, it is now well-recognized that the immune system plays a major role in breast cancer development and progression, first by eradicating malignant cells and then by gradually becoming redirected by cancer cells, locally as well as systemically, to promote cancer growth and metastasis [16]. The recent success of immune checkpoint inhibition treatments in other cancer types has sparked interest in its use also in breast cancer. Checkpoint inhibition has already been shown to be effective in triple-negative breast cancer (TNBC), with multiple clinical trials ongoing, as reviewed recently [17]. The United States Food and Drug Administration (FDA) has recently approved pembrolizumab, which has previously been used for metastatic disease, for treatment of high-risk early-stage TNBC [18]. But even with the growing knowledge of the important local effects of the immune system within the tumor microenvironment (TME), little is still known about the systemic immunity, including immune-modulatory factors in blood, which is highly relevant in the metastatic setting. A better understanding of the immunological responses in MBC will give important information regarding the disease development and progression and has potential to improve treatment of MBC.

The aim of this study was to evaluate a broad panel of blood-borne immuno-oncology markers in MBC patients and their respective relevance for prediction of survival (OS and PFS).

## Patients and methods

### Patients and study design

**Fig. 1:**
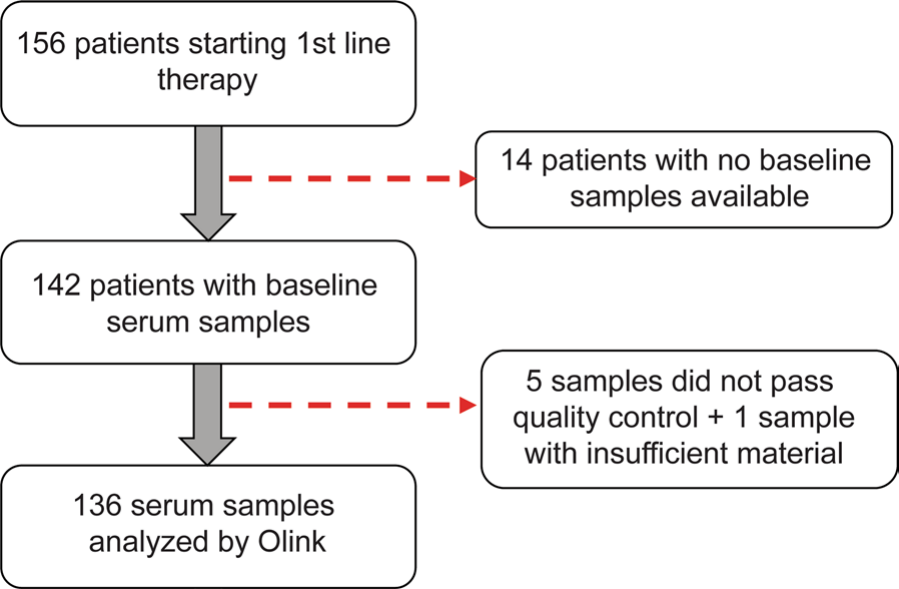
Flowchart of study cohort and serum samples

Patients with newly diagnosed MBC were enrolled into a prospective observational trial (ClinicalTrials.gov NCT01322893) conducted at Skåne university hospital and Halmstad county hospital, Sweden, between April 2011 and June 2016. Briefly, the inclusion criteria were MBC diagnosis, age ≥ 18 years, Eastern Cooperative Oncology Group (ECOG) performance status score of 0–2, and a predicted life expectancy of more than two months. Exclusion criteria included previous systemic therapy for metastatic disease or other malignant disease diagnosis within the last five years, and inability to understand the study information. The study was approved by the regional research ethics committee at Lund University (Dnr 2010/135) and conducted in accordance with the Declaration of Helsinki. Written informed consent was obtained from all patients prior to study inclusion. The cohort constitutes 156 patients with newly diagnosed MBC, planned for first-line systemic treatment and has previously been described in detail [19–21]. Serum samples were taken at baseline (before start of systemic therapy) and out of the 156 patients, 20 serum samples were lost or excluded (for 14 patients; no serum sample was available, five samples did not pass the quality control and one sample did not provide enough material) (Fig. 1). The number of circulating tumor cells (CTCs) and presence of CTC clusters were evaluated using the CellSearch™ system, and the results have been reported in a previous study [19].

### Biochemical analysis

Serum samples were collected at baseline (before start of systemic treatment). The samples were collected in serum tubes, centrifugated, aliquoted and stored at -80 °C until analysis. The proteomic analyses were performed at the Olink facility in Uppsala, Sweden, using the OLINK Proteomics PEA technology [11, 22] (Olink Target 96 Immuno-Oncology panel, analyzing 92 protein biomarkers). Patient information was blinded to personnel at Olink Bioscience and samples were distributed randomly into the analysis plates. Samples were processed at Olink Bioscience according to their manuals, including quality control of data, and normalization of measured results. Intensity normalization was used to adjust for inter-plate variability, with the plate median as the normalization factor. Results were provided as relative values; normalized protein expression (NPX), an arbitrary unit on a log_2_ scale. On this scale, a one-unit increase of the NPX value corresponds to a doubling of the protein content, with high NPX values corresponding to a high protein concentration. NPX values can, however, not be converted to absolute protein concentrations. Nor are the NPX values directly comparable between different serum proteins, as they are calculated separately for individual analytes. Data were pre-processed as follows: Proteins with NPX values below the limit of detection (LOD) in more than 15% of the samples and samples with > 15% proteins below LOD were removed from further analysis. For the remaining values, we rescaled the NPX values by subtracting the LOD except for those already below two standard deviations of the LOD, which in turn were assigned as not applicable (NA). All missing values were then imputed based on a nearest neighbor approach (kNN imputation with k = 5).

### Statistical analyses for feature selection

Random forest (RF) analyses and random survival forest (RSF) analyses were performed to identify predictors of overall survival (OS) or progression-free survival (PFS). OS was calculated from the time of baseline blood sampling to death from any cause. PFS was calculated from the time of baseline blood sampling to progression evaluated using modified RECIST criteria, as described previously [19]. If the outcome was not reached, the time variables were censored at the last follow-up. To remove potential biasing effects that established clinical prognostic factors may have, we regressed the effect of the following factors through multi-linear modeling prior to RF/RSF: age, ECOG, metastasis-free interval, number of metastases, site of metastasis, breast cancer subtype, NHG and number of CTCs (> / ≤ 5 CTCs). RF was initially employed for parameter selection, i.e., to identify serum proteins with little informative value in predicting OS/PFS, from which we used the top 20 proteins as input for further parameter tuning, as indicated for the RSF below. RSF is a random forest method that is used for analysis of right-censored survival data. The method identifies the variables that best predict survival, OS and PFS, respectively. For RSF, we performed the analyses in a three-step process as follows: First, hyperparameter tuning was performed for maximum number of features, number of total estimators, minimum sample number per leaf, and minimum number of samples per split through a randomized grid search process coupled with fivefold cross-validation, repeated 100 times. Second, with the best fit hyperparameters, we then performed tenfold cross-validation for final model training and prediction and repeated this process 100 times. Third, with the best selected parameters and final trained model, we identified feature importance by permutation importance to assess how removal of different features affected the model performance [23]. Recursive feature elimination was then used to examine how many features were needed to maximize the model concordance, Harrell’s C-statistic, for both PFS and OS. We further validated the selected serum proteins using both Cox regression and Penalized Cox regression, both with regressed and un-regressed data. Machine learning technique analyses (RF, RSF, Cox regression and penalized Cox regression) were performed using R 4.1, Rstudio 1.1.456, limma 3.50.0, mixomics 6.16.0, jupyter 1.0.0, scikit-learn 0.24.2, scikit-survival 0.15.0.post0 and python 3.9.9.

### Cox regression analyses and correlations to patient and tumor characteristics

Cox regression analyses were performed for estimation of hazard ratios (HR) with 95% confidence interval (CI) for both PFS and OS according to the continuous serum protein NPX values, both univariable analyses and multivariable analyses adjusted for the prognostic factors mentioned above. The association between serum protein levels and different patient and tumor characteristics was analyzed using Fisher’s exact test or logistic regression where appropriate, with continuous serum protein NPX values dichotomized using the median value for each protein as the cut-off point. The study was performed according to the REMARK criteria [24].

All *P* values presented are two-sided and should in general be regarded as continuous measures of evidence, but following Benjamin et al*.*, two thresholds are used throughout this paper: suggestive evidence for *P* values between 0.05 and 0.005 and significant evidence for *P* < 0.005 [25]. In addition, false discovery rate (FDR) was used to adjust Cox regression *P* values for multiple testing. *P* values for all 92 proteins studied were used to calculate so-called *q* values separately for uni- and multivariable analysis and for each endpoint. SPSS Statistics version 27.0.1.0 (IBM, Armonk, NY, USA) was used for correlation and Cox regression analyses. FDR analyses, as described in Benjamini and Hoffberg [26], were carried out using the user contributed program qqvalue.ado in Stata 17.0, (StataCorp LLC, College Station, TX, USA).

## Results

### Patient characteristics

A total of 156 patients with newly diagnosed metastatic breast cancer (MBC) were enrolled in the study and serum samples were collected at baseline, before start of systemic therapy. Among the 156 patients, serum samples were not available for 20 patients (Fig. 1). Information on the whole patient cohort has been published before [19]. Patient and tumor characteristics for the 136 patients included in this study, summarized in Table 1, are representative for the whole cohort. Twenty-eight patients were diagnosed with de novo MBC and 108 patients were diagnosed with distant recurrence. The median follow-up time from baseline was 20 months (range 0–66) for patients alive at the last medical visit. Median age of the patients at time of MBC diagnosis was 65 years (range 40–90 years). Breast cancer subtype was determined in metastases first-hand and primary tumors second-hand, with 94 patients (69%) having estrogen receptor-positive (ER +) and human epidermal growth factor receptor 2 negative (HER2-) tumors, 15 patients (11%) had HER2 + (ER ±) tumors, and 24 patients (18%) had triple-negative breast cancer (TNBC), with the subtype missing for two patients. Eighty patients (59%) had visceral metastases (defined as lung, liver, brain, peritoneal, and/or pleural involvement).

**Table 1:** Clinicopathological variables in metastatic breast cancer patients at baseline

	Included (136)	%	Excluded (20)	%
*Age, median (range)*	65 (40–90)		65 (45–82)	
< 50	65	48	10	50
≥ 50	71	52	10	50
*Baseline ECOG*				
0	75	57	16	90
1	36	27	1	5
2	21	16	1	5
Missing	4		2	
*PT NHG*				
I-II	68	62	10	71
III	42	38	4	29
Missing	26		6	
*PT tumor size*				
1	49	38	8	44
2	46	36	5	28
3	17	13	3	17
4	17	13	2	11
Missing	7		2	
*PT node status*				
negative	39	33	5	28
positive	79	67	13	72
missing	18		2	
*Breast cancer subtype*				
ER + HER2-	94	71	11	61
HER2 +	15	11	5	28
ER- HER2-	24	18	2	11
Missing	3		2	
*Metastasis-free interval (years)*				
0	28	21	3	15
> 0–3	25	18	3	15
≥ 3	83	61	14	70
Missing	0		0	
*Metastatic sites, number*				
< 3	96	71	13	65
≥ 3	40	29	7	35
Missing	0		0	
*Site of metastasis*				
Non-Visceral	56	41	9	45
Visceral	80	59	11	55
Missing	0		0	
*1st line of treatment for MBC*				
Endocrine	55	43	3	19
Chemotherapy	60	47	11	69
HER2-targeted	13	10	2	12
Missing	8		4	
*CTC*				
< 5	65	49	8	44
≥ 5	69	51	10	56
Missing	2		2	
*One or more CTC clusters*				
No	108	81	14	78
Yes	26	19	4	22
Missing	2		2	

*CTC* circulating tumor cells; *ECOG* Eastern Cooperative Oncology Group; *ER* estrogen receptor; *HER2* human epidermal growth factor; *MBC* metastatic breast cancer; *NHG* Nottingham histological grade; *PT* primary tumor

### Feature selection with random forest

In order to identify novel immuno-oncology markers that predict survival in MBC patients, we analyzed the levels of 92 serum proteins from 136 patients with newly diagnosed MBC using multiplex proximity extension assay (PEA; the Olink Target 96 Immuno-Oncology panel). Due to the large number of serum proteins measured for each MBC patient, the likelihood for false positive findings can be inflated. To identify the proteins with highest importance for survival analyses, we first used random forest (RF) analyses for an unbiased selection of variables of importance for PFS and OS and eliminated the 25% of proteins with the lowest median importance for PFS and OS. Additional File 1: Fig. S1 shows feature importance after the RF analyses, sorted according to OS feature importance (top) and PFS feature importance (bottom).

### Random survival forest to select top proteins for prediction of survival

**Fig. 2:**
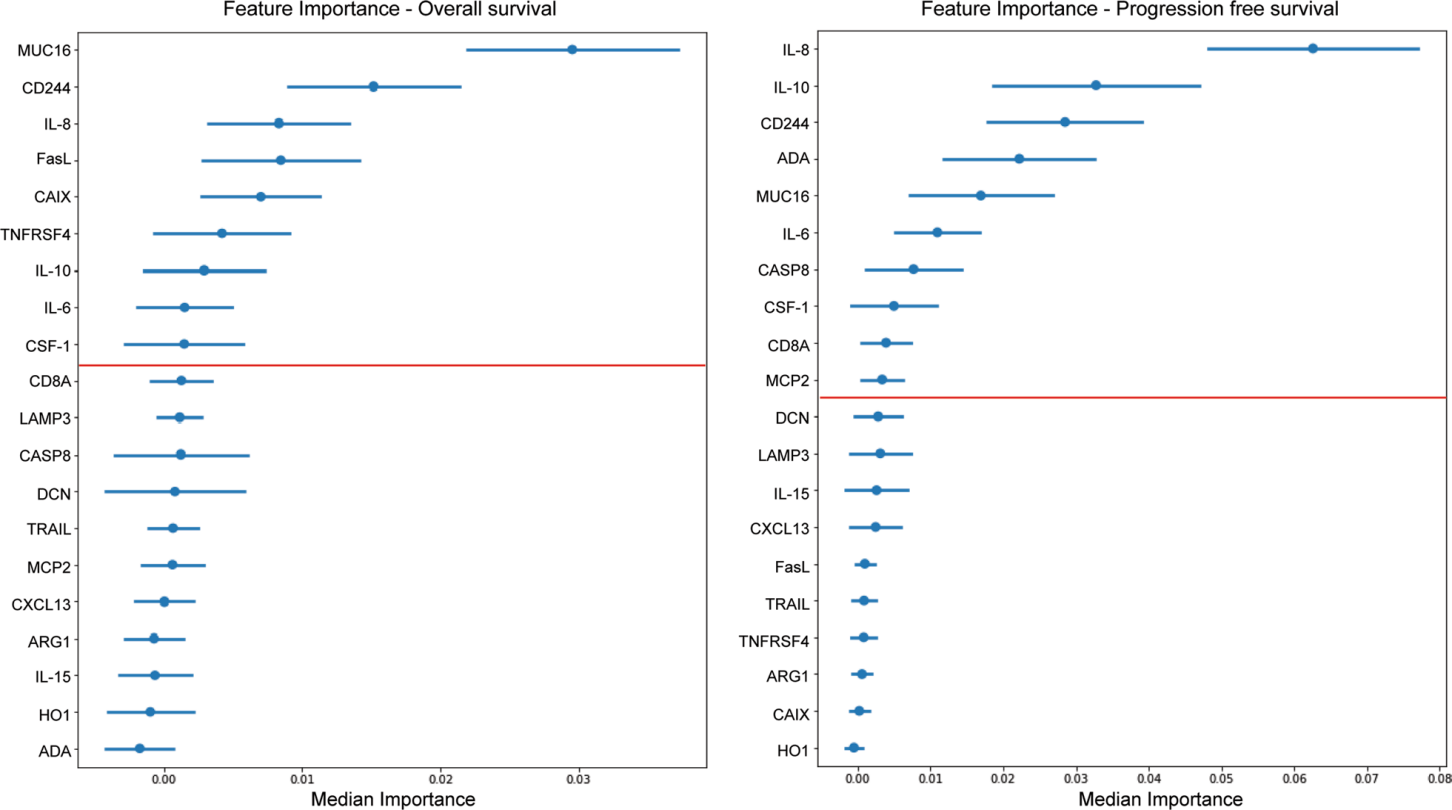
Top 20 serum proteins arranged by overall feature importance for prediction of survival. Random survival forest (RSF) analyses used to select the top 20 proteins with highest importance for prediction of overall survival (OS, left) and progression-free survival (PFS, right). Red line shows the cut-off of serum proteins that lead to highest median score of model concordance for OS (left) and PFS (right)

Using the top 20 proteins for prediction of survival, identified using RF (Additional File 1: Fig. S1), we next used random survival forest (RSF) (Fig. 2) to further select the proteins of highest relevance. Throughout, we employed Harrell's concordance index (c-index) to quantify RSF model concordance with PFS or OS and considering the right-censoring of the data. For OS, the median training and test C-indices were 0.689 and 0.630, respectively, with Mucin-16 (MUC16) showing a higher overall importance than other proteins, followed by natural killer cell receptor 2B4 (CD244), interleukin (IL) 8, Fas antigen ligand (FasL), and carbonic anhydrase IX (CAIX). The 20 serum proteins with highest overall importance can be seen in Fig. 2 (left). For PFS, the median training and test C-indices were 0.727 and 0.600, respectively, with the top five variables being similar to the top five variables for OS. IL-8 shows the highest overall importance, followed by IL-10, CD244, Adenosine Deaminase (ADA), and MUC16 (Fig. 2 (right)).

To examine how many features were needed to maximize the model concordance, we utilized recursive feature elimination by considering the median feature importance from the RSF, removing the features one by one and parameterizing the models, using cross-validation, and computing the median score for all models. This identified the number of features that lead to the highest median score to be considered. For OS, the top nine variables of highest predictive importance were: MUC16, CD244, IL-8, FasL, CAIX, tumor necrosis factor receptor superfamily member 4 (TNFRSF4), IL-10, IL-6, and macrophage colony-stimulating factor 1 (CSF-1) (Fig. 2, left). The top ten variables for predicting PFS were: IL-8, IL-10, CD244, ADA, MUC16, IL-6, Caspase-8 (CASP8), CSF-1, T-cell surface glycoprotein CD8 alpha chain (CD8A) and monocyte chemotactic protein 2 (MCP2) (Fig. 2, right). Five of the top nine proteins for predicting OS were also found within the top ten proteins for predicting PFS, comprising in total 13 proteins identified as being most relevant for prediction of outcome.

### Confirming top proteins using penalized cox regression

To further confirm the proteins of highest importance, we used penalized Cox regression by employing elastic nets, where variable weight on the model is identified for individual variables. Penalized Cox regression (Additional File 2: Fig. S2) indicated that the top variables for both OS and PFS are similar to those observed using RSF, further supporting the relevance of these proteins. Similarly, the results of penalized Cox regression on un-regressed data were in accordance with the results from the RSF analyses (Additional File 3: Fig. S3 and data not shown). Additional File 4: Table S1 and Additional File 5: Table S2 show how the top 13 proteins selected were ranked for prediction of OS and PFS, respectively, in each model used (RF, Cox regression, RSF, penalized Cox regression and penalized Cox regression on un-regressed data). These results indicate a high degree of concordance between the models used in identifying the serum proteins of highest importance for predicting survival.

### Associations between serum proteins and survival

To further determine the potential of the identified 13 serum proteins to predict OS or PFS, we next quantified the association (HR, Hazard Ratio) and the evidence for an association (*P* value) between the serum levels (NPX values) of the proteins of highest importance, as selected using RSF, and survival using unadjusted and adjusted Cox regression analysis with either OS or PFS as end point (Table 2). For OS, all the nine evaluated serum proteins showed suggestive or significant evidence for correlation with survival in both uni- and multivariable analysis, with the exception of CD244 that showed a weak correlation with survival in multivariable analysis (Table 2). A twofold increase in the serum level of FasL was significantly associated with improved OS (UV: HR = 0.58, 95%CI: (0.40–0.83), *P* = 0.0031; MV: HR = 0.47, 95%CI: (0.30–0.75), *P* = 0.0013), while a doubling of the levels of CD244 showed evidence of being associated with improved OS (UV: HR = 0.57, 95%CI: (0.35–0.95), *P* = 0.030). Elevated levels of CAIX, IL-8 and IL-10 all showed significant association with worse OS in both uni- and multivariable analysis (Table 2). Interestingly, CSF-1 displayed a high HR (UV: HR = 11.16, 95% CI: (4.12–30.20); MV: HR = 6.05, 95% CI: (1.59–23.00)) for a twofold increase in uni- and multivariable analysis, respectively, compared to the other serum proteins (HR around 1.0).

**Table 2:** Cox regression Hazard ratios for serum protein levels, in relation to survival

	Overall survival Hazard ratio (95%CI)						Progression-free survival Hazard ratio (95%CI)					
Serum protein	Unadjusted	*P* value	*q* value	Adjusted	*P* value	*q* value	Unadjusted	*P* value	*q* value	Adjusted	*P* value	*q* value
ADA	–	–	–	–	–	–	2.13 (1.46–3.09)	< 0.0001	0.0067	2.39 (1.38–4.16)	0.0020	0.1703
CASP8	–	–	–	–	–	–	1.65 (1.28–2.14)	0.00012	0.0099	1.73 (1.25–2.39)	0.00087	0.0786
CAIX	1.56 (1.27–1.93)	< 0.0001	0.0017	1.44 (1.15–1.81)	0.0018	0.1399	–	–	–	–	–	–
CD244	0.57 (0.35–0.95)	0.030	0.9216	0.74 (0.37–1.49)	0.40	0.9818	0.50 (0.31–0.79)	0.0028	0.2250	0.50 (0.27–0.91)	0.022	0.9819
CD8A	–	–	–	–	–	–	0.91 (0.68–1.22)	0.55	0.9899	0.97 (0.64–1.46)	0.88	0.9819
CSF-1	11.16 (4.12–30.20)	< 0.0001	0.0002	6.05 (1.59–23.00)	0.0083	0.5976	4.90 (2.24–10.68)	< 0.0001	0.0059	5.00 (1.61–15.54)	0.0055	0.4584
FasL	0.58 (0.40–0.83)	0.0031	0.1862	0.47 (0.30–0.75)	0.0013	0.1060	–	–	–	–	–	–
IL-6	1.37 (1.21–1.56)	< 0.0001	< 0.0001	1.54 (1.14–2.10)	0.0056	0.4126	1.26 (1.11–1.43)	0.00029	0.0245	1.47 (1.11–1.93)	0.0068	0.5677
IL-8	1.47 (1.31–1.66)	< 0.0001	< 0.0001	1.63 (1.33–1.98)	< 0.0001	0.0002	1.35 (1.20–1.53)	< 0.0001	0.0001	1.44 (1.18–1.76)	0.00034	0.0310
IL-10	1.41 (1.19–1.66)	< 0.0001	0.0058	1.42 (1.14–1.77)	0.0019	0.1492	1.32 (1.11–1.56)	0.0015	0.1176	1.35 (1.11–1.63)	0.0025	0.2114
MCP2	–	–	–	–	–	–	0.89 (0.68–1.16)	0.39	0.9899	0.88 (0.63–1.24)	0.47	0.9819
MUC16	1.40 (1.22–1.61)	< 0.0001	0.0001	1.27 (1.05–1.55)	0.015	0.9818	1.23 (1.08–1.39)	0.0014	0.1157	1.22 (1.02–1.45)	0.029	0.9819
TFNSFR4	1.78 (1.29–2.46)	0.00043	0.0307	1.99 (1.23–3.23)	0.0054	0.4062	–	–	–	–	–	–

The hazard ratio corresponds to a doubling of normalized protein expression values (NPX values), an arbitrary unit on a log2 scale

*q* values are *P* values adjusted for multiple comparison (all 92 serum proteins) using false discovery rate (FDR)

– denotes not relevant HRs since these are not top proteins selected for the respective outcome

*CI* confidence interval

For PFS, eight out of ten analyzed top serum proteins showed suggestive or significant evidence for association with PFS in both uni- and multivariable analyses (Table 2). Again, elevated levels of CD244 showed evidence for association with improved PFS (UV: HR = 0.50, 95%CI: (0.31–0.79), *P* = 0.0028; MV: HR = 0.50, 95%CI (0.27–0.91), *P* = 0.022). Higher serum levels of ADA, CASP8, IL-8 and IL-10 all showed significant association with worse PFS in both uni- and multivariable analysis (Table 2). In addition, FDR analysis was used to adjust Cox regression *P* values for multiple testing. *P* values for all 92 proteins studied were used to calculate so-called *q* values separately for UV and MV analyses for OS as well as PFS. Corresponding *q* values for all *P* values of the top 13 proteins are shown in Table 2, illustrating that IL-8 shows the strongest evidence for correlation to OS and PFS in both UV and MV analyses after FDR correction (OS: UV, *q* < 0.0001; MV, *q* = 0.0002; PFS: UV, *q* = 0.0001; MV, *q* = 0.0310). Regarding the other identified top proteins CAIX (*q* = 0.0017), CSF-1 (*q* = 0.0002), IL-6 (*q* < 0.0001), MUC16 (*q* = 0.0001), IL-10, (*q* = 0.0058) and TFNSFR4 (*q* = 0.0307) all showed strong evidence for correlation to OS in UV analyses after FDR correction, whereas the evidence for correlation in adjusted MV analysis was weaker (CAIX, *q* = 0.1399; CSF-1, *q* = 0.5976; IL-6, *q* = 0.4126; MUC16 *q* = 0.9818; IL-10, *q* = 0.1492; TFNSFR4, *q* = 0.4062) (Table 2). For correlations to PFS, ADA (q = 0.0067), CASP8 (*q* = 0.0099), CSF-1 (*q* = 0.0059) and IL-6 (*q* = 0.0245) all showed strong evidence for correlation in UV analyses after FDR correction, whereas the evidence for correlation in adjusted MV analyses were weaker (ADA, *q* = 0.1703; CASP8, *q* = 0.0786; CSF-1, *q* = 0.4584; IL-6, *q* = 0.5677 and IL-10, *q* = 0.2114).

### Serum protein correlations with clinicopathological features

To investigate the potential correlation of serum proteins with clinicopathological features, the continuous protein levels were dichotomized into high or low levels using the respective median as the cut-off point (Table 3). Eight out of 13 serum proteins showed evidence for correlation with ECOG performance status. High levels of CD244 and FasL showed evidence for correlation with better ECOG performance status whereas high CSF-1, IL-8 and MUC16 all correlated significantly with worse ECOG performance status. Nine out of 13 proteins showed suggestive or significant evidence for correlation with increased number of metastatic sites, with higher levels of IL-6 and MUC16 correlating significantly with > 3 metastatic sites (Table 3). Interestingly, higher levels of ADA, IL-8, IL-10 and MUC16 all correlated significantly with the presence of CTC clusters, with ADA and MUC16 further correlating with high numbers of CTCs (≥ 5), and IL-8 showing evidence for correlating with high levels of CTCs. All correlations to clinicopathological features and serum proteins are summarized in Table 3.

**Table 3:** Odds ratios of top serum protein levels by patient and tumor clinicopathological features

				ADA			CAIX			CASP8			CD244			CD8A	
Clinicopathological features		*N*	OR	95%CI	*P* value	OR	95%CI	*P* value	OR	95%CI	*P* value	OR	95%CI	*P* value	OR	95%CI	*P* value
Age	< 65	65	1			1			1			1			1		
	≥ 65	71	0.52	0.26–1.03	0.086^a^	1.06	0.54–2.08	1.00^a^	0.94	0.48–1.85	1.00^a^	0.94	0.48–1.85	1.00^a^	0.59	0.30–1.16	0.17^a^
ECOG	0	75	1			1			1			1					
	1	36	1.27	0.57–2.82	0.55^b^	1.14	0.51–2.53	0.75^b^	3.36	1.46–7.75	0.005^b^	0.83	0.37–1.84	0.65^b^	0.78	0.35–1.73	0.54^b^
	2	21	2.55	0.92–7.03	0.071^b^	3.18	1.11–9.10	0.031^b^	3.36	1.21–9.32	0.020^b^	0.33	0.12–0.95	0.039^b^	1.07	0.41–2.82	0.89^b^
NHG	I-II	68	1			1			1			1					
	III	42	1.53	0.71–3.32	0.33^a^	1.06	0.49–2.29	1.00^a^	1.15	0.53–2.49	0.84^a^	0.81	0.37–1.75	0.7^a^	0.59	0.27–1.29	0.24^a^
PT tumor size	1	49	1			1			1			1					
	2	46	1.88	0.83–4.25	0.13b	1.05	0.47–2.34	0.91^b^	1.03	0.46–2.31	0.94^b^	1.03	0.46–2.31	0.94^b^	1.23	0.55–2.75	0.62^b^
	3	17	1.40	0.46–4.27	0.55^b^	0.52	0.17–1.64	0.27^b^	1.09	0.36–3.30	0.88^b^	3.99	1.14–13.98	0.031^b^	1.38	0.46–4.17	0.57^b^
	4	17	2.90	0.92–9.13	0.070^b^	1.08	0.36–3.26	0.89^b^	2.25	0.72–7.06	0.16^b^	1.75	0.57–5.36	0.33^b^	2.25	0.72–7.06	0.16^b^
PT node status	Neg	39	1			1			1			1					
	Pos	79	0.84	0.39–1.80	0.70^a^	0.65	0.30–1.40	0.33^a^	0.55	0.26–1.20	0.17^a^	1.62	0.75–3.53	0.24^a^	1.47	0.68–3.18	0.43^a^
MFI	0	28	1			1			1			1					
	0–3	25	1.27	0.43–3.76	0.66^b^	1.73	0.58–5.16	0.33^b^	1.10	0.37–3.26	0.86^b^	1.78	0.59–5.36	0.31^b^	1.08	0.37–3.19	0.88^b^
	> 3	83	0.93	0.40–2.19	0.87^b^	1.07	0.46–2.53	0.87^b^	0.77	0.33–1.81	0.55^b^	0.84	0.36–1.99	0.70^b^	0.98	0.41–2.30	0.96^b^
Metastatic sites	< 3	96	1			1			1			1					
	≥ 3	40	2.39	1.11–5.13	0.038^a^	1.33	0.63–2.79	0.57^a^	2.79	1.28–6.05	0.014^a^	1	0.48–2.09	1.00^a^	1.33	0.63–2.79	0.57^a^
Site of metastasis	Non-visceral	56	1			1			1			1					
	Visceral	80	1.28	0.64–2.53	0.60^a^	2.37	1.18–4.78	0.023^a^	1.63	0.82–3.24	0.22^a^	1.13	0.57–2.24	0.86^a^	1.13	0.57–2.24	0.86^a^
First line	Endocrine	55	1			1			1			1					
treatment	Chemotherapy	60	3.08	1.44–6.62	0.004^b^	1.98	0.94–4.17	0.072^b^	2.14	1.01–4.52	0.047^b^	0.72	0.35–1.51	0.39^b^	1.19	0.57–2.47	0.65^b^
	HER2 targeted	13	4.63	1.25–17.07	0.021^b^	2.59	0.75–8.98	0.13^b^	2.80	0.81–9.73	0.11^b^	0.66	0.20–2.23	0.51^b^	0.46	0.13–1.68	0.24^b^
CTC	< 5	65	1			1			1			1					
	≥ 5	69	3.44	1.69–7.00	0.001^a^	1.27	0.65–2.51	0.50^a^	1.62	0.82–3.21	0.17^a^	0.62	0.31–1.22	0.17^a^	1.20	0.61–2.36	0.73^a^
CTC clusters	Neg	108	1			1			1			1					
	Pos	26	4.49	1.67–12.08	0.002^a^	2.27	0.93–5.55	0.082^a^	2.27	0.93–5.55	0.082^a^	0.54	0.22–1.29	0.19^a^	1.21	0.51–2.86	0.83^a^
PAM50 subtype	Luminal A	39	1			1			1			1					
	Luminal B	41	6.25	2.35–16.55	0.0002^b^	1.13	0.46–2.78	0.78^b^	2.56	1.03–6.33	0.043^b^	1.83	0.75–4.44	0.18^b^	0.90	0.37–2.17	0.81^b^
	HER2 enriched	13	6.53	1.64–25.93	0.0077^b^	3.60	0.94–13.79	0.062^b^	4.50	1.16–17.41	0.029^b^	1.11	0.31–3.91	0.87^b^	0.38	0.10–1.45	0.16^b^
	Basal like	14	2.90	0.81–10.33	0.10^b^	4.00	1.06–15.08	0.041^b^	3.60	1.00–12.95	0.050^b^	1.29	0.38–4.40	0.68^b^	0.86	0.25–2.91	0.81^b^

				CSF1			FasL			IL-6			IL-8			IL-10	
Clinicopathological features		*N*	OR	95%CI	*P* value	OR	95%CI	*P* value	OR	95%CI	*P* value	OR	95%CI	*P* value	OR	95%CI	*P* value
Age	< 65	65	1			1			1			1			1		
	≥ 65	71	1.34	0.68–2.64	0.49^a^	0.66	0.34–1.30	0.30^a^	1.19	0.61–2.34	0.73^a^	0.84	0.43–1.64	0.73^a^	1.19	0.61–2.34	0.73^a^
ECOG	0	75	1			1			1			1			1		
	1	36	3.77	1.63–8.74	0.002^b^	0.42	0.19–0.96	0.039^b^	2.97	1.30–6.78	0.010^b^	2.50	1.11–5.64	0.027^b^	1.68	0.75–3.74	0.20^b^
	2	21	4.71	1.63–13.59	0.004^b^	0.33	0.12–0.92	0.034^b^	2.73	1.01–7.40	0.049^b^	19.00	4.10–88.10	< 0.001^b^	2.69	0.97–7.42	0.057^b^
NHG	I-II	68	1			1			1			1			1		
	III	42	1.24	0.57–2.67	0.70^a^	0.63	0.29–1.36	0.33^a^	1.39	0.64–3.02	0.44^a^	1.45	0.67–3.13	0.43^a^	1.02	0.47–2.21	1.00^a^
PT tumor size	1	49	1			1			1			1			1		
	2	46	1.59	0.71–3.57	0.26^b^	1.04	0.46–2.32	0.93^b^	1.46	0.65–3.27	0.36^b^	1.46	0.65–3.28	0.36^b^	0.95	0.42–2.13	0.90^b^
	3	17	0.93	0.31–2.86	0.90^b^	2.71	0.83–8.87	0.099^b^	0.93	0.31–2.86	0.90^b^	1.09	0.36–3.30	0.88^b^	1.62	0.53–4.94	0.40^b^
	4	17	2.44	0.78–7.68	0.13^b^	1.27	0.42–3.84	0.67^b^	3.20	0.98–10.49	0.055^b^	1.38	0.46–4.17	0.57^b^	1.62	0.53–4.94	0.40^b^
PT node status	Neg	39	1			1			1			1			1		
	Pos	79	0.88	0.41–1.90	0.85^a^	1.2	0.55–2.58	0.7^a^	1.03	0.48–2.22	1.00^a^	0.76	0.35–1.63	0.56^a^	0.80	0.37–1.72	0.70^a^
MFI	0	28	1			1			1			1			1		
	0–3	25	1.50	0.50–4.46	0.47^b^	1.73	0.58–5.16	0.33^b^	0.82	0.28–2.46	0.73^b^	1.10	0.37–3.26	0.86^b^	0.58	0.19–1.72	0.33^b^
	> 3	83	0.89	0.38–2.09	0.78^b^	1.07	0.46–2.53	0.87^b^	0.52	0.22–1.25	0.14^b^	0.77	0.33–1.81	0.55^b^	0.93	0.40–2.20	0.87^b^
Metastatic sites	< 3	96	1			1			1			1			1		
	≥ 3	40	2.79	1.28–6.05	0.014^a^	0.87	0.42–1.82	0.85^a^	3.27	1.49–7.19	0.004^a^	2.79	1.28–6.05	0.014^a^	2.39	1.11–5.13	0.038^a^
Site of metastasis	Non-visceral	56	1			1			1			1			1		
	Visceral	80	2.09	1.04–4.19	0.055^a^	1.28	0.64–2.53	0.60^a^	2.70	1.33–5.47	0.009^a^	1.85	0.92–3.68	0.12^a^	1.63	0.82–3.24	0.22^a^
First line	Endocrine	55	1			1			1			1			1		
treatment	Chemotherapy	60	1.59	0.76–3.33	0.22^b^	0.71	0.34–1.49	0.37^b^	1.85	0.88–3.89	0.11^b^	1.85	0.88–3.89	0.11^b^	1.71	0.82–3.60	0.15^b^
	HER2 targeted	13	1.62	0.48–5.47	0.44^b^	0.2	0.049–0.81	0.024^b^	1.89	0.56–6.39	0.31^b^	1.89	0.56–6.39	0.31^b^	1.29	0.38–4.34	0.69^b^
CTC	< 5	65	1			1			1			1			1		
	≥ 5	69	1.83	0.92–3.63	0.088^a^	1	0.51–1.97	1.00^a^	1.44	0.73–2.84	0.31^a^	2.07	1.04–4.12	0.04^a^	1.52	0.77–3.01	0.30^a^
CTC clusters	Neg	108	1			1			1			1			1		
	Pos	26	1.52	0.64–3.62	0.39^a^	0.8	0.34–1.88	0.67^a^	1.04	0.44–2.44	1.00^a^	8	2.58–24.82	< 0.001^a^	4.33	1.61–11.63	0.004^a^
PAM50 subtype	Luminal A	39	1			1			1			1			1		
	Luminal B	41	2.02	0.83–4.95	0.12^b^	1.34	0.56–3.24	0.51^b^	1.23	0.51–2.97	0.64^b^	2.28	0.93–5.61	0.072^b^	1.11	0.46–2.67	0.81^b^
	HER2 enriched	13	1.00	0.28–3.52	1.00^b^	0.47	0.12–1.78	0.27^b^	1.51	0.43–5.33	0.52^b^	4.02	1.04–15.46	0.043^b^	1.36	0.39–4.79	0.63^b^
	Basal like	14	0.88	0.26–3.00	0.83^b^	0.79	0.23–2.70	0.71^b^	2.33	0.66–8.24	0.19^b^	4.46	1.18–16.90	0.028^b^	2.92	0.78–10.91	0.11^b^

				MCP2			MUC16			TNFRSF4	
Clinicopathological features		*N*	OR	95%CI	*P* value	OR	95%CI	*P* value	OR	95%CI	*P* value
Age	< 65	65	1			1			1		
	≥ 65	71	1.34	0.68–2.64	0.49^a^	1.19	0.61–2.34	0.73^a^	1.06	0.54–2.08	1.00^a^
ECOG	0	75	1			1			1		
	1	36	1.60	0.72–3.57	0.25^b^	1.77	0.80–3.96	0.16^b^	1.68	0.75–3.74	0.20^b^
	2	21	1.04	0.39–2.74	0.94^b^	5.08	1.68–15.35	0.004^b^	2.18	0.81–5.89	0.12^b^
NHG	I-II	68	1			1			1		
	III	42	0.94	0.44–2.04	1.00^a^	0.96	0.45–2.08	1.00^a^	1.06	0.49–2.29	1.00^a^
PT tumor size	1	49	1			1			1		
	2	46	0.75	0.33–1.67	0.48^b^	0.88	0.39–1.96	0.75^b^	2.05	0.91–4.65	0.085^b^
	3	17	0.72	0.24–2.19	0.57^b^	1.49	0.49–4.55	0.49^b^	1.78	0.58–5.40	0.31^b^
	4	17	0.92	0.30–2.77	0.88^b^	1.17	0.39–3.54	0.78^b^	2.90	0.92–9.13	0.070^b^
PT node status	Neg	39	1			1			1		
	Pos	79	1.39	0.65–3.01	0.44^a^	1.26	0.58–2.72	0.70^a^	1.33	0.61–2.87	0.056^a^
MFI	0	28	1			1			1		
	0–3	25	1.07	0.36–3.14	0.91^b^	1.73	0.58–5.16	0.33^b^	1.18	0.50–2.79	0.70^b^
	> 3	83	1.24	0.53–2.93	0.62^b^	1.07	0.46–2.53	0.87^b^	0.95	0.39–2.31	0.90^b^
Metastatic sites	< 3	96	1			1			1		
	≥ 3	40	2.39	1.11–5.13	0.038^a^	3.27	1.49–7.19	0.004^a^	2.79	1.28–6.05	0.014^a^
Site of metastasis	Non-visceral	56	1			1			1		
	Visceral	80	2.37	1.18–4.78	0.023^a^	2.09	1.04–4.19	0.055^a^	1.63	0.82–3.24	0.22^a^
First line	Endocrine	55	1			1			1		
treatment	Chemotherapy	60	0.83	0.40–1.74	0.63^b^	2.17	1.02–4.60	0.044^b^	1.58	0.76–3.30	0.22^b^
	HER2 targeted	13	0.37	0.10–1.35	0.13^b^	4.26	1.16–15.68	0.029^b^	0.81	0.23–2.78	0.74^b^
CTC	< 5	65	1			1			1		
	≥ 5	69	0.74	0.38–1.46	0.49^a^	3.44	1.69–7.00	< 0.001^a^	1.52	0.77–3.01	0.30^a^
CTC clusters	Neg	108	1			1			1		
	Pos	26	0.56	0.23–1.34	0.28^a^	5.88	2.06–16.76	< 0.001^a^	3.39	1.32–8.74	0.015^a^
PAM50 subtype	Luminal A	39	1			1			1		
	Luminal B	41	0.99	0.41–2.39	0.99^b^	1.84	0.76–4.46	0.18^b^	1.83	0.75–4.44	0.18^b^
	HER2 enriched	13	0.54	0.15–1.93	0.34^b^	1.68	0.47–5.93	0.42^b^	0.81	0.22–2.92	0.75^b^
	Basal like	14	0.86	0.25–2.91	0.81^b^	3.59	0.96–13.50	0.058^b^	2.33	0.66–8.24	0.19^b^

*CTC* circulating tumor cells; *ECOG* Eastern Cooperative Oncology Group; *HER2* human epidermal growth factor receptor 2; *MFI* metastasis-free interval; *NHG* Nottingham histological grade; *PT* primary tumor

^a^*P* value from Fisher’s exact test

^b^*P* value from logistic regression

## Discussion

MBC is considered an incurable disease with limited treatment strategies and novel biomarkers to improve prognostication are urgently needed. Recent advances in immune therapies, and expanding knowledge on the role of the immune system in cancer progression, has sparked interest in its use in breast cancer. Checkpoint inhibitors are already in use in TNBC patients and have potential to be implemented also in treatment of other MBC subtypes. However, not only does the immune system have a localized effect within the TME, but secreted and systemic components such as chemokines and cytokines play a role in cell trafficking and metastasis of tumor cells [27]. Analyzing protein biomarkers in peripheral blood samples constitute a fast, cheap, and easily accessible approach for prognostication as well as monitoring treatment response. In this study, we aimed to identify novel serum proteins that could serve as biomarkers for survival of MBC patients. We found that out of 92 serum proteins analyzed, 13 were of high importance for prediction of survival and 11 of these were independent prognostic factors after adjusting for established clinical prognostic factors. After strict correction for multiple comparisons using FDR analyses, IL-8 still showed strong evidence for correlation to both OS and PFS, whereas the evidence for correlation to survival was weaker for the other top proteins.

Whereas a handful of studies have investigated serum markers in early breast cancer and association to outcome of different treatments, only limited information is available for MBC [2–4]. Furthermore, biomarker studies in breast cancer patients have in the past used a limited panel of serum proteins, [7–9]. With the emerging multiplex screening technology, it is now easier than ever to screen hundreds or even thousands of proteins from smaller sample volumes [11]. The use of PEA technology provides researchers with an accurate method with high sensitivity to measure proteins, but studies that have used this method for screening of BC patients have thus far not focused specifically on serum proteins from MBC patients [12, 13]. We therefore aimed to fill the knowledge gap and identify serum proteins in MBC patients that could provide prognostic information.

When analyzing the top ranked serum proteins of importance for prediction of OS and PFS, several display high median importance. Out of the top nine proteins selected for association with OS, all showed suggestive or significant evidence for correlation in traditional multivariable Cox regression analyses. For PFS, eight out of the top ten serum proteins showed suggestive or significant evidence for correlation using multivariable Cox regression analyses. After FDR correction for multiple comparisons, IL-8 still showed strong evidence for correlation to both OS and PFS.

Elevated serum levels of IL-8 and IL-10 both showed significant correlation to worse OS and PFS in both uni- and multivariable analyses. In line with their association with worse survival, high levels of IL-8 and IL-10 further correlated with features associated with more aggressive disease, such as more metastatic sites and presence of CTC clusters. However, after FDR correction, IL-8 still showed strong evidence for correlation to OS and PFS, whereas the prognostic evidence for IL-10 levels was weaker.

IL-8 has previously been reported to promote breast cancer progression through induction of cell invasion and angiogenesis, as previously reviewed [28]. Serum levels of IL-8 are associated with larger tumor size and increased number of metastases in MBC patients, as well as being predictive of long-term survival in MBC patients that have received chemotherapy [4, 10, 29]. Elevated serum levels of IL-10 have been found in breast cancer patients compared to healthy controls, and IL-10 serum levels correlated with cancer stage [30, 31]. To our knowledge, serum IL-10 has not previously been shown to be an independent prognostic marker for survival in MBC, only in combination with other serum cytokines [31, 32]. In our study, elevated serum levels of IL-10 were shown to be an independent prognostic factor for both worse OS and PFS in multivariable analysis. After strict FDR correction for multiple comparisons, there was still evidence for correlation to OS in UV analyses whereas the evidence for correlation in MV analyses was weaker.

Similarly, we observed that elevated serum levels of CAIX showed significant correlation with worse OS in both uni- and multivariable analyses, in agreement with its association with visceral metastases and with what has previously been shown for MBC patients [33–35]. After FDR correction, there was still strong evidence for correlation to OS in UV analyses, whereas the evidence for correlation to OS in MV analysis was weaker. CAIX inhibition has been evaluated as a therapeutic target for triple-negative breast cancer and MBC using various breast cancer models, with several clinical trials for patients with solid tumors, including MBC, ongoing [36, 37]. In our study cohort, high serum levels of CSF-1, IL-6, MUC16 and TNFRSF4 all associated with more metastatic sites. Moreover, for PFS, elevated serum levels of CSF-1 and IL-6 showed suggestive evidence for correlation to worse survival, which is in accordance with previous studies [7, 38]. After FDR correction for multiple comparisons, strong evidence for correlations to OS and PFS were still seen in UV analyses, whereas the evidence for correlations to OS in MV analyses were weaker. Higher serum levels of MUC16 and TNFRSF4 further showed evidence for correlating with worse PFS and OS. MUC16 has in combination with other serum tumor markers been shown to be associated with survival but has to our knowledge not previously been shown to be an independent factor after adjusting for all other clinically used prognostic factors in previously untreated MBC[5, 6]. After FDR correction, MUC16 and TFNSFR4 both showed evidence for correlation to OS in UV analyses, whereas the evidence for correlations to OS in MV analyses were weaker. There are very few studies on serum TNFRSF4 and its role in MBC, with studies showing that the levels of TNFRSF4 are elevated in patients with breast cancer, but to our knowledge, no studies have been published on serum levels of TNFRSF4 and association with survival in MBC patients.

Elevated serum levels of ADA showed a significant correlation to worse PFS in both uni- and multivariable analyses. After FDR correction, evidence for correlation to PFS was observed in UV analyses, whereas the evidence for correlation to PFS in MV analysis was weaker. Interestingly, we further saw a significant correlation with the PAM50 HER2-enriched (HER2E) subtype and features usually associated with worse disease including more metastatic sites and CTCs. Previously, elevated levels of ADA in plasma have been linked to pro-tumoral M2-like macrophage polarization, with elevated serum levels of ADA correlating with elevated serum levels of soluble CD163 [39]. Defects in ADA have been linked to impaired M2 macrophage polarization [40, 41]. ADA has also been shown to be secreted by monocytes and macrophages, but the exact mechanism for its possible role in M2-like polarization is yet to be discovered [42, 43]. Further, we did not find any studies mentioning a possible connection between ADA and HER2 + breast cancer subtype in the literature, indicating that, to our knowledge, our study would be the first to possibly link elevated ADA expression to the HER2 + BC subtype.

Similarly, high serum levels of CASP8 correlated significantly with worse PFS in uni- and multivariable analyses, which is in accordance with its association with more metastatic sites. After FDR correction for multiple comparisons, the evidence for correlation to PFS was strong in UV analysis but slightly weaker in MV analysis. A previous study found that caspase-3 expression, but not caspase-8, in breast cancer tissue was associated with worse survival in early-stage breast cancer patients with hormone receptor positive, and non-basal like subtype BC [44]. Higher caspase-8 levels in blood or serum have been correlated to worse outcome for patients with spontaneous intracerebral hemorrhage [45] and sepsis [46]. But, to our knowledge, caspase-8 has not previously been associated with outcome for cancer patients.

Out of the top 13 serum proteins identified for correlation to survival, only two showed suggestive or significant evidence for correlation with improved OS and PFS, CD244 and FasL. In many different cancer types, CD244 is viewed as an anti-inflammatory and pro-tumor receptor, with CD244 inhibition suggested as a possible therapy to overcome checkpoint inhibitor resistance [47]. In our study, however, higher levels of serum CD244 showed evidence for association with better OS, as well as significantly better PFS in univariable analysis. However, after strict correction for multiple comparisons using FDR, CD244 showed weaker evidence for correlations to OS and PFS. CD244 mRNA levels have been found to be downregulated in breast cancer tissue [48]. To our knowledge, serum CD244 has only previously been studied in patients with non-small cell lung cancer, where elevated CD244 serum levels is a negative prognostic marker [49]. In addition, serum levels of FasL have been shown to be elevated after chemotherapy compared to levels before chemotherapy in stage II and III breast cancer patients, indicating FasL as a possible marker for evaluating treatment efficacy in breast cancer [50]. However, elevated plasma levels of Fas, the receptor for FasL, have been associated with worse survival for primary, recurring, and metastatic breast cancer patients [8, 51]. Combined, our results indicate a novel correlation between elevated serum levels of CD244 and FasL in MBC patients and improved survival.

One of the main strengths of this study is the combination of multiple machine learning techniques and more traditional statistical methods, to optimize selection of variables and estimation of prognostic effects in a well-characterized cohort of MBC. Through a combination of RF and RSF, together with cross-validation, we removed uninformative features and identified those that yield the highest risk concordance. Using the Olink’s multiplex PEA provided a wide selection of immuno-oncology proteins to analyze. Another strength is that the study collected serum samples before start of therapy, rendering results for a population of untreated MBC patients where most other cohorts are including MBC patients on different lines of therapy. The size of the MBC patient cohort can be regarded as a limitation, with serum samples from 136 patients analyzed and 92 different proteins evaluated. Hence, the results from this study need to be validated in another independent cohort of MBC patients. Currently, serum samples are being collected from newly diagnosed MBC patients in another prospective observational study in our center (SCAN-B-rec, NCT03758976) that will serve as a validation cohort. However, the results from the present study highlight the potential of specific immuno-oncology proteins for prognostication in newly diagnosed MBC patients.

## Conclusion

In the present study we show that by analyzing a broad panel of immuno-oncology markers in serum from MBC patients, we identified several proteins that significantly predicted OS and PFS, respectively. Importantly, these markers carried prognostic information independently of other established prognostic factors including CTCs. After strict correction of multiple comparisons using FDR, IL-8 still showed strong evidence for predicting both OS and PFS. The results need to be confirmed in an independent cohort, and additional analyses of follow-up samples during treatment could be evaluated to further elucidate the potential of these markers for monitoring of therapy response. These results further highlight the relevance of the systemic immune response in patients with MBC.

## Supplementary Information

**Additional file 1.** **Figure S1.** Serum protein feature importance scores for survival prediction. Random forest analyses used to rank serum proteins by importance for predicting survival. Importance score scale organized in order of importance for overall survival (OS; blue dots) shown in upper panel and importance score scale organized in order of importance for progression-free survival (PFS; red dots) shown in lower panel for each serum protein.

**Additional file 2.** **Figure S2.** Cox penalized regression using elastic nets, identifying variable weights among the top variables for overall survival (left) and progression-free survival (right).

**Additional file 3.** **Figure S3.** Cox penalized regression using elastic nets with unregressed data, for overall survival (left) and progression-free survival (right).

**Additional file 4.** **Table S1.** Ranking score from each model used, for top 9 serum proteins predicting overall survival (OS)

**Additional file 5.** **Table S2.** Ranking score from each model used, for top 10 serum proteins predicting progression-free survival (PFS)

## Data Availability

The datasets used and analyzed during the current study are available from the corresponding author on reasonable request.

## Abbreviations

BC: Breast cancer
CI: Confidence interval
CTC: Circulating tumor cell
ECOG: Eastern Cooperative Oncology Group
ER: Estrogen receptor
FDA: Food and Drug Administration
HER2: Human epidermal receptor 2
HR: Hazard ratio
kNN: K-nearest neighbors algorithm
LOD: Limit of detection
MBC: Metastatic breast cancer
MFI: Metastasis-free interval
MV: Multivariable
NA: Not applicable
NHG: Nottingham histological grade
NPX: Normalized protein expression
OR: Odds ratio
OS: Overall survival
PEA: Proximity extension assay
PFS: Progression-free survival
PT: Primary tumor
RECIST: Response evaluation criteria in solid tumors
REMARK: REporting recommendations for tumor MARKer
RF: Random forest
RSF: Random survival forest
RT-qPCR: Real-time quantitative polymerase chain reaction
TME: Tumor microenvironment
TNBC: Triple-negative breast cancer
UV: Univariable
