# Supplementary material for: Serum immuno-oncology markers carry independent prognostic information in patients with newly diagnosed metastatic breast cancer, from a prospective observational study
Source: Breast Cancer Res. 2023 Mar 21;25:29. doi: 10.1186/s13058-023-01631-6 (PMC10031935; doi:10.1186/s13058-023-01631-6)
Supplement: Supplementary file 1 — Additional file 1. Figure S1. Serum protein feature importance scores for survival prediction. Random forest analyses used to rank serum proteins by importance for predicting survival. Importance score scale organized in order of importance for overall survival (OS; blue dots) shown in upper panel and importance score scale organized in order of importance for progression-free survival (PFS; red dots) shown in lower panel for each serum protein. [file 13058_2023_1631_MOESM1_ESM.pdf]

Additional File 1

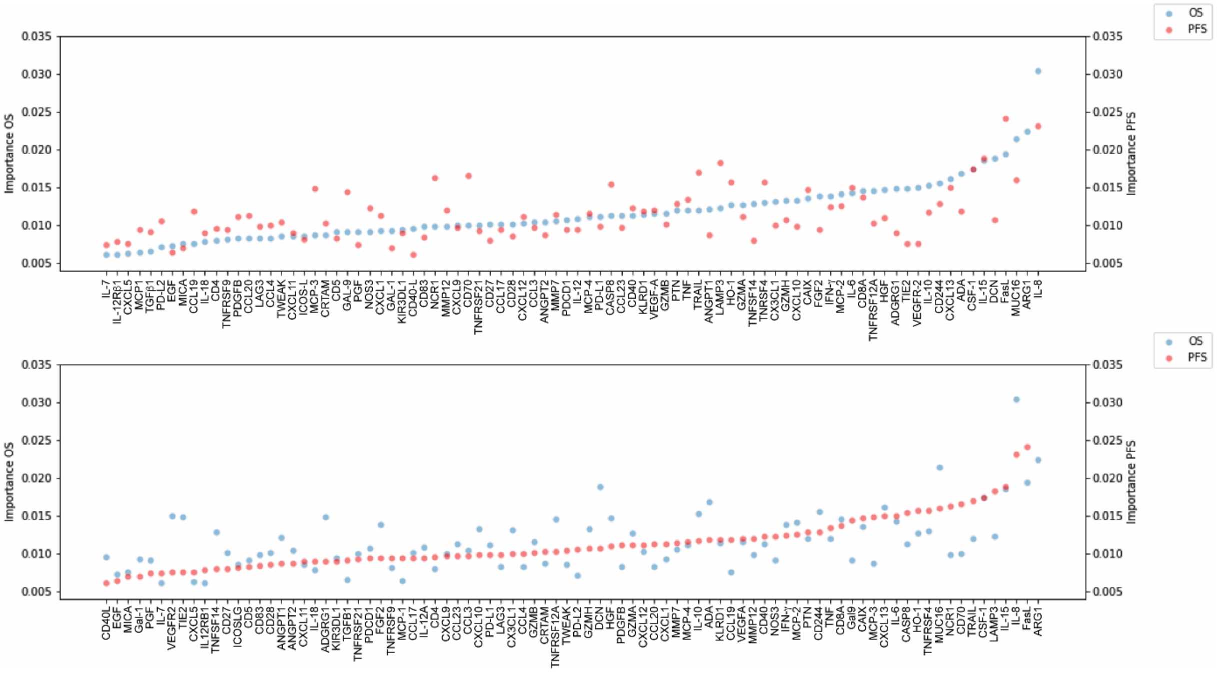

Supplementary figure 1. Serum protein feature importance scores for survival prediction. Random forest analyses used to rank serum proteins by importance for predicting survival. Importance score scale organized in order of importance for overall survival (OS; blue dots) shown in upper panel and importance score scale organized in order of importance for progression-free survival (PFS; red dots) shown in lower panel for each serum protein.
