# Supplementary material for: Serum immuno-oncology markers carry independent prognostic information in patients with newly diagnosed metastatic breast cancer, from a prospective observational study
Source: Breast Cancer Res. 2023 Mar 21;25:29. doi: 10.1186/s13058-023-01631-6 (PMC10031935; doi:10.1186/s13058-023-01631-6)
Supplement: Supplementary file 3 — Additional file 3. Figure S3. Cox penalized regression using elastic nets with unregressed data, for overall survival (left) and progression-free survival (right). [file 13058_2023_1631_MOESM3_ESM.pdf]

Additional File 3

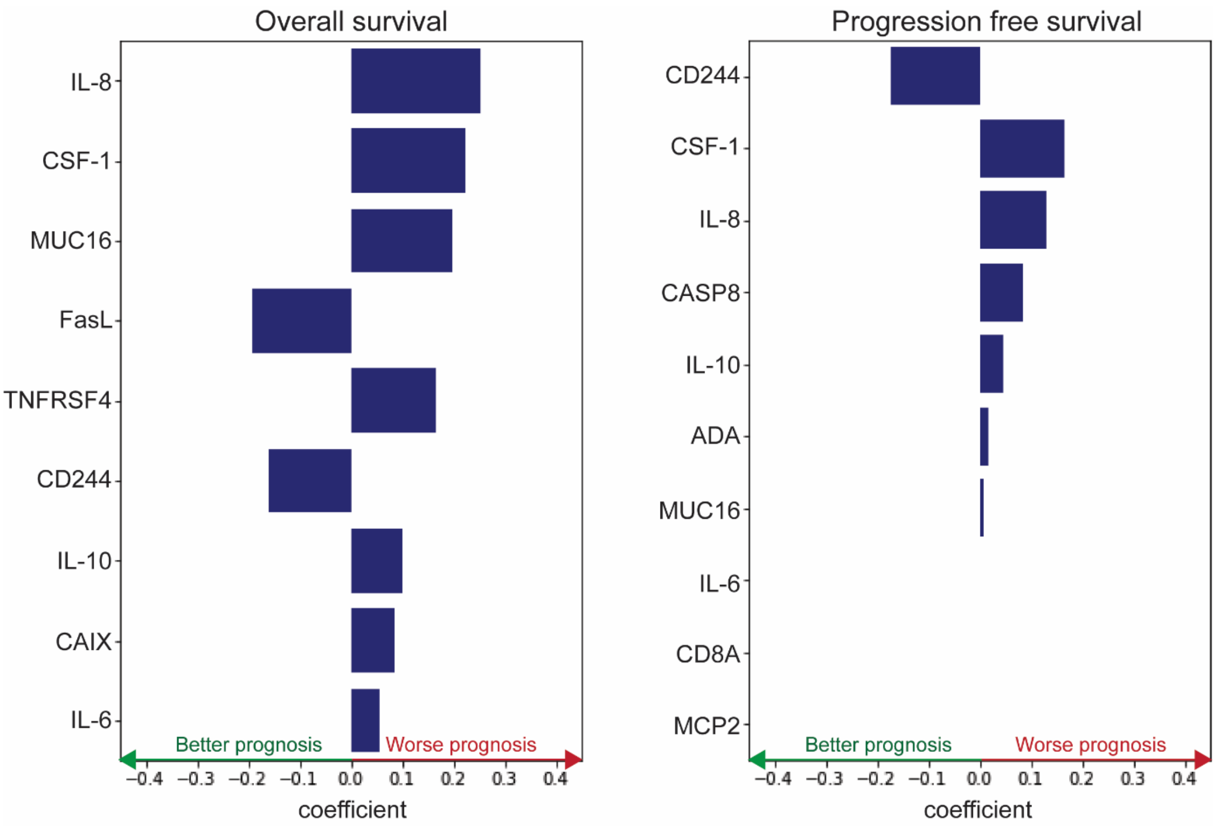

Supplementary figure 3. Cox penalized regression using Elastic Nets with unregressed data, for overall survival (left) and progression free survival (right).
