# Supplementary material for: Serum immuno-oncology markers carry independent prognostic information in patients with newly diagnosed metastatic breast cancer, from a prospective observational study
Source: Breast Cancer Res. 2023 Mar 21;25:29. doi: 10.1186/s13058-023-01631-6 (PMC10031935; doi:10.1186/s13058-023-01631-6)
Supplement: Supplementary file 4 — Additional file 4. Table S1. Ranking score from each model used, for top 9 serum proteins predicting overall survival (OS) [file 13058_2023_1631_MOESM4_ESM.pdf]

Additional File 4

Supplementary table 1. Ranking score from each model used, for top 9 serum proteins predicting OS

Protein	RF	CR	RSF	CPR	CPRU
CAIX	22	16	5	8	8
CD244	10	45	2	2	6
CSF-1	7	4	10	6	2
FasL	4	24	4	5	4
IL-6	18	5	9	9	9
IL-8	1	2	3	1	1
IL-10	11	7	7	3	7
MUC16	3	1	1	7	3
TFNSFR4	26	20	6	4	5

RF, Random Forest; CR, Cox Regression; RSF, Random Survival Forest; CPR, Cox Penalized Regression; CPRU, Cox Penalized Regression Unregressed.
