# Supplementary material for: Serum immuno-oncology markers carry independent prognostic information in patients with newly diagnosed metastatic breast cancer, from a prospective observational study
Source: Breast Cancer Res. 2023 Mar 21;25:29. doi: 10.1186/s13058-023-01631-6 (PMC10031935; doi:10.1186/s13058-023-01631-6)
Supplement: Supplementary file 5 — Additional file 5. Table S2. Ranking score from each model used, for top 10 serum proteins predicting progression-free survival (PFS) [file 13058_2023_1631_MOESM5_ESM.pdf]

Additional File 5

Supplementary table 2. Ranking score from each model used, for top 10 serum proteins predicting PFS

Protein	RF	CR	RSF	CPR	CPRU
ADA	31	13	4	6	6
CASP8	13	5	7	4	4
CD244	21	9	3	1	1
CD8A	19	55	9	9	9
CSF-1	6	8	8	3	2
IL-6	14	7	6	8	8
IL-8	3	1	1	2	3
IL-10	32	14	2	5	5
MCP2	23	71	10	10	10
MUC16	10	2	5	7	7

RF, Random Forest; CR, Cox Regression; RSF, Random Survival Forest; CPR, Cox Penalized Regression; CPRU, Cox Penalized Regression Unregressed.
